# *MuMag2022*: a software tool for analyzing magnetic field dependent unpolarized small-angle neutron scattering data of bulk ferromagnets

**DOI:** 10.1107/S1600576722005349

**Published:** 2022-07-28

**Authors:** Michael P. Adams, Mathias Bersweiler, Elizabeth M. Jefremovas, Andreas Michels

**Affiliations:** a Department of Physics and Materials Science, University of Luxembourg, 162A Avenue de la Faïencerie, L-1511 Luxembourg, Grand Duchy of Luxembourg; b Departamento CITIMAC, Facultad de Ciencias, Universidad de Cantabria, 39005 Santander, Spain; Oak Ridge National Laboratory, USA, and North Carolina State University, USA

**Keywords:** small-angle neutron scattering, micromagnetism, magnetic materials, nanocomposites

## Abstract

The MATLAB-based software tool *MuMag2022* is presented for the analysis of magnetic-field-dependent unpolarized small-angle neutron scattering (SANS) data of bulk ferromagnets such as elemental nanocrystalline ferromagnets, magnetic nanocomposites or magnetic steels. On the basis of the micromagnetic theory for the magnetic SANS cross section, the program analyzes unpolarized total (nuclear and magnetic) SANS data within the approach-to-saturation regime. The main features of *MuMag2022* are the estimation of the exchange-stiffness constant, and of the strength and spatial structure of the magnetic anisotropy field and the magnetostatic field due to longitudinal magnetization fluctuations. *MuMag2022* is open source and available as a standalone executable for Windows at https://mumag.uni.lu.

## Abstract 2

The MATLAB-based software tool *MuMag2022* is presented for the analysis of magnetic-field-dependent unpolarized small-angle neutron scattering data of bulk ferromagnets such as elemental nanocrystalline ferromagnets, magnetic nanocomposites or magnetic steels.

## 1. Introduction

Magnetic small-angle neutron scattering (SANS) is in many respects different from nonmagnetic nuclear SANS or small-angle X-ray scattering (SAXS). This is mainly related to the following points: (i) the quantity of interest in magnetic SANS is the three-dimensional magnetization vector field of the sample, 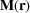 , while it is the scalar nuclear density 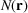 that is of relevance in nonmagnetic SANS. Therefore, besides changes in the magnitude of 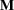 , spatial variations in the orientation of 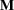 are of special importance for magnetic SANS. (ii) The method for obtaining 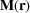 , a continuum micromagnetic variational ansatz aiming to minimize the total magnetic energy of the system, is conceptually different from that used to obtain 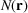 – mostly concepts based on particle form factors and structure factors. (iii) As a consequence of the quantum-mechanical exchange interaction, magnetization profiles are smoothly varying continuous functions of the position, which entails the absence of sharp (discontinuous) features in the magnetic microstructure. Although models with a smoothly varying 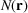 have also been developed for nonmagnetic SANS (*e.g.* Schmidt *et al.*, 1991 ▸; Heinemann *et al.*, 2000 ▸), the most widespread approach in particle scattering is to fit a certain form-factor model, implying the presence of a sharp interface, to a set of experimental data. These differences have fundamental consequences regarding the scattering behavior; *e.g.* magnetic SANS on bulk ferromagnets does generally not exhibit an asymptotic 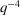 Porod law, but may reveal larger power-law exponents (*e.g.* Bersweiler *et al.*, 2021 ▸). Related to the previous statement is the fact that the correlation function of magnetic systems exhibits a different functional dependency from the density–density autocorrelation function of nonmagnetic particle systems.

A theoretical framework for magnetic SANS has been developed in recent years (Michels, 2021 ▸), which allows one to analyze the momentum-transfer and applied-field dependence of the total unpolarized SANS cross section within the approach-to-saturation regime of the macroscopic magnetization. This approach provides information on the magnetic interaction parameters such as the exchange-stiffness constant, and the strength and spatial structure of the magnetic anisotropy and magnetostatic field. The software tool *MuMag2022* presented here encodes the relevant expressions and allows for the analysis of ( 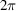 azimuthally averaged) magnetic-field-dependent unpolarized SANS data of bulk ferromagnets; examples are elemental nanocrystalline ferromagnets, magnetic nanocomposites or magnetic steels.

The article is organized as follows: Section 2 summarizes, for the two most often employed scattering geometries, the main theoretical expressions for the unpolarized nuclear and magnetic SANS cross section and explains the data analysis procedure. Section 3 provides some details on the operation of the *MuMag2022* software and Section 4 presents some selected example cases.

## 2. Magnetic SANS theory – unpolarized neutrons

The magnetic-field-dependent SANS of bulk ferromagnets is typically dominated by the spin-misalignment scattering, *i.e.* the part of the magnetic SANS cross section that is related to the transverse magnetization Fourier coefficients. Since the spin-misalignment SANS is independent of the polarization of the incident neutron beam, half-polarized (‘spin-up’ and ‘spin-down’) SANSPOL^1^ experiments, which additionally provide access to nuclear–magnetic interference terms, do not provide significantly more information regarding spin misalignment than can already be learned from the analysis of the unpolarized scattering. Chiral correlations are also ignored in our treatment. Therefore, the first version of our software package *MuMag2022* considers only the case of unpolarized SANS. In the following, we summarize the main equations for the nuclear and magnetic SANS cross section of bulk ferromagnets, focusing on the two most often used scattering geometries which have the externally applied magnetic field either perpendicular or parallel to the incoming beam.

### 2.1. **k**_0_ ⊥ **H**_0_

**Figure 1:**
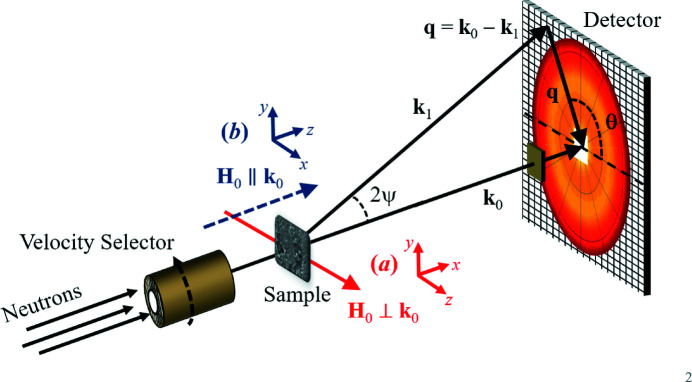
Sketch of the two most often employed scattering geometries in magnetic SANS experiments. (*a*) 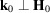 ; (*b*) 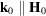 . We emphasize that in both geometries the applied-field direction 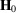 defines the 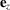 direction of a Cartesian laboratory coordinate system. The momentum transfer or scattering vector 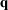 corresponds to the difference between the wavevectors of the incident ( 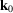 ) and the scattered ( 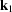 ) neutrons, *i.e.* 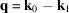 . Its magnitude for elastic scattering, 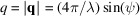 , depends on the mean wavelength λ of the neutrons and on the scattering angle 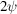 . SANS is usually implemented as elastic scattering ( 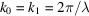 ), and the component of 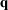 along the incident neutron beam [*i.e.* 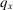 in (*a*) and 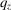 in (*b*)] is neglected. The angle θ specifies the orientation of the scattering vector on the two-dimensional detector; θ is measured between 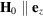 and 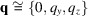 (*a*) and between 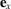 and 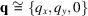 (*b*). Note that in many SANS publications the scattering angle is denoted by the symbol 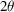 . However, in order to comply with our previous notation (see *e.g.* the publications in the reference list), we prefer to denote this quantity by 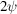 .

For the scattering geometry where the applied magnetic field 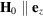 is perpendicular to the wavevector 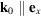 of the incoming neutron beam [see Fig. 1 ▸(*a*)], the elastic (unpolarized) SANS cross section 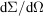 at scattering vector 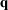 can be written as (Michels, 2021 ▸)

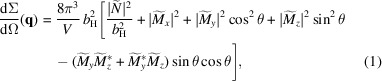

where *V* is the scattering volume, 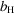 = 2.91 × 10^8^ A^−1^ m^−1^ is the magnetic scattering length, 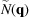 and 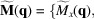 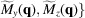 denote, respectively, the Fourier transforms of the nuclear scattering length density and of the magnetization 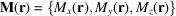 , and θ represents the angle between 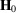 and 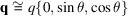 ; the asterisks 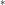 mark the complex-conjugated quantity.

As shown by Honecker & Michels (2013 ▸), near magnetic saturation, 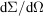 can be evaluated by means of micromagnetic theory. In particular, 

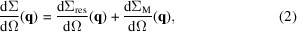

where 

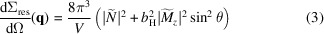

represents the nuclear and magnetic residual SANS cross section, which is measured at complete magnetic saturation (infinite field), and 

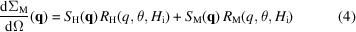

is the spin-misalignment SANS cross section. The magnetic scattering due to transverse spin components, with related Fourier amplitudes 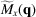 and 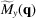 , is contained in 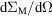 , which decomposes into a contribution 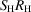 due to perturbing magnetic anisotropy fields and a part 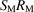 related to magnetostatic fields. The micromagnetic SANS theory considers a uniform exchange interaction and a random distribution of the magnetic easy axes, as is appropriate for a statistically isotropic polycrystalline ferromagnet (Michels, 2021 ▸). Spatial variations in the magnitude of the saturation magnetization are explicitly taken into account via the function 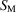 (see below). Moreover, in the approach-to-saturation regime it is assumed that 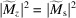 , where 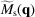 denotes the Fourier transform of the saturation magnetization profile 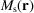 .

Regarding the decomposition of the SANS cross section [equation (2)], we emphasize that it is 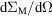 that depends on the magnetic interactions (exchange, anisotropy, magnetostatics), while 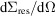 is determined by the geometry of the underlying grain microstructure (*e.g.* the particle shape or the particle-size distribution). If in a SANS experiment the approach-to-saturation regime can be reached for a particular magnetic material (as is assumed here), then the residual SANS can be obtained by an analysis of field-dependent data via the extrapolation to infinite field (see Section 2.4). In a sense, for a bulk ferromagnet, the scattering at saturation resembles the topographical background in Kerr-microscopy experiments, which needs to be subtracted in order to access the magnetic domain structure of the sample (McCord & Hubert, 1999 ▸).

The anisotropy-field scattering function (in units of cm^−1^) 

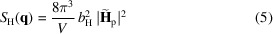

depends on 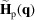 , which represents the Fourier transform of the spatial structure of the magnetic anisotropy field 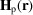 of the sample, whereas the scattering function of the longitudinal magnetization (in units of cm^−1^) 

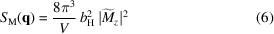

provides information on the spatial variation of the saturation magnetization 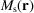 ; for instance, in a multiphase magnetic nanocomposite, 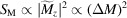 , where 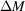 denotes the jump of the magnetization magnitude at internal (particle–matrix) interfaces. Note that the volume average of 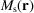 equals the macroscopic saturation magnetization 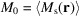 of the sample, which can be measured with a magnetometer. The corresponding dimensionless micromagnetic response functions can be expressed as (Michels, 2021 ▸)

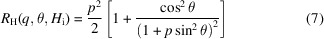

and 

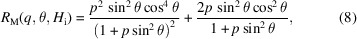

where 

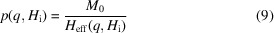

is a dimensionless function and θ represents the angle between 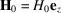 and 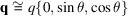 . The effective magnetic field 

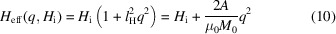

depends on the internal magnetic field 

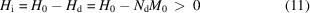

and on the micromagnetic exchange length of the field 

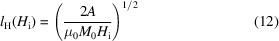

( 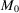 saturation magnetization; *A* exchange-stiffness parameter; 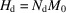 demagnetizing field; 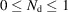 demagnetizing factor; 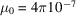 Tm A^−1^). Note that 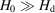 in the approach-to-saturation regime. The θ dependence of 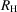 and 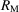 arises essentially as a consequence of the magnetodipolar interaction. Depending on the values of *q* and 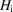 , a variety of angular anisotropies may be seen on a two-dimensional position-sensitive detector (Michels, 2021 ▸).

The effective magnetic field 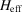 [equation (10)] consists of a contribution due to the internal field 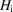 and the exchange field 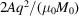 . An increase of 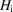 increases the effective field only at the smallest *q* values, whereas 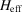 at larger *q* is always very large (∼10–100 T) and independent of 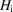 (Michels, 2021 ▸). The latter statement may be seen as a manifestation of the fact that exchange forces tend to dominate on small length scales (Aharoni, 2000 ▸). Since 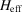 appears predominantly in the denominators of the final expressions for 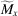 and 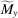 [compare equations (3.68) and (3.69) of Michels (2021 ▸)], its role is to suppress the high-*q* Fourier components of the magnetization, which correspond to sharp real-space fluctuations. On the other hand, long-range magnetization fluctuations, at small *q*, are effectively suppressed when 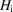 is increased.

By assuming that the functions 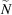 , 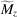 and 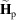 depend only on the magnitude 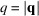 of the scattering vector, one can perform an azimuthal average of equation (2), *i.e.* 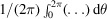 . The resulting expressions for the response functions then read

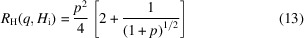

and 

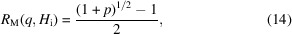

so that the azimuthally averaged total nuclear and magnetic SANS cross section can be written as

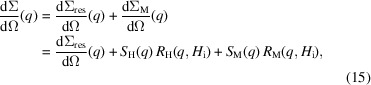

where 

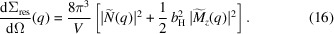

For materials exhibiting a uniform saturation magnetization (*e.g.* single-phase materials), the magnetostatic scattering contribution 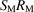 [to 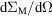 , compare equation (4)] is expected to be much smaller than the anisotropy-field-related term 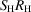 [compare *e.g.* Fig. 23 of Michels (2014 ▸)].

We emphasize that the micromagnetic theory behind the *MuMag2022* software results in an analytical expression for the two-dimensional SANS cross section as a function of the magnitude *q* and the orientation θ of the scattering vector 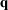 . These analytical expressions can be azimuthally averaged over the full angular detector range 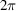 (or any other range) and compared with correspondingly averaged experimental SANS data; in other words, it is not required that the experimental input SANS data are isotropic.

### 2.2. **k**_0_ ∥ **H**_0_

For the scattering geometry where the external magnetic field 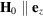 is parallel to the incident-beam direction 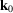 [see Fig. 1 ▸(*b*)], the total azimuthally averaged SANS cross section can be written as (Michels, 2021 ▸)

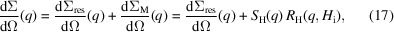

where the residual SANS cross section explicitly reads 

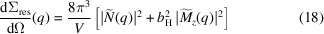

and the response function is isotropic (*i.e.* θ independent), 

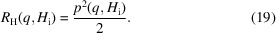




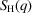
 is given by equation (5), and we note that in this geometry 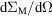 does not depend on 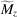 fluctuations and equals the expression for the single-phase material case (Michels, 2021 ▸). In other words, the possible two-phase (particle–matrix-type) nature of the underlying microstructure is (for 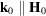 ) only contained in 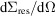 , and not in 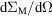 .

### 2.3. Mean-square anisotropy and magnetostatic field

Numerical integration of 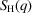 and 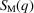 over the whole 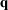 space, *i.e.*


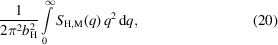

yields, respectively, the mean-square anisotropy field 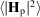 and the mean-square longitudinal magnetization fluctuation 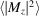 (Michels, 2021 ▸). These quantities are, respectively, defined as 

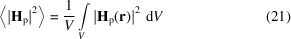

and 

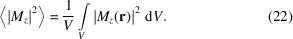

Equation (20) follows from equations (21) and (22) by using Parseval’s theorem of Fourier theory and the definitions of 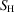 and 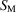 [equations (5) and (6)]. Since experimental data for 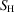 and 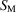 are only available within a finite range of momentum transfers between 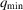 and 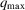 (see Fig. 5 below), one can only obtain rough lower bounds for these quantities. Therefore, the numerical integration of equation (20) is carried out for 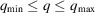 ; 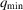 denotes the first experimental data point, while 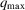 is defined by equation (24) below.

Knowledge of 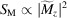 and of the residual SANS cross section 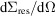 [equations (16) and (18)] allows one to obtain the nuclear scattering 

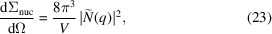

without using sector-averaging procedures (in unpolarized scattering) or polarization analysis (Honecker *et al.*, 2010 ▸).

### 2.4. Neutron data analysis procedure

**Figure 2:**
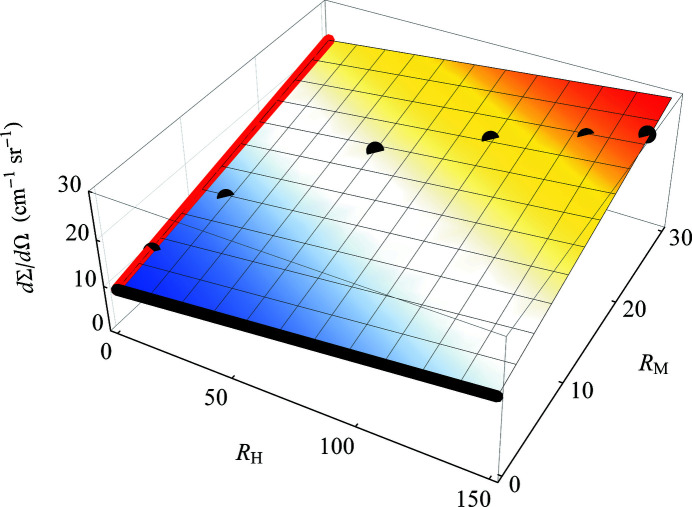
Illustration of the neutron data analysis procedure according to equation (15). The total 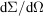 (solid circles) of the the iron-based alloy Nanoperm is plotted at 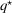 = 0.114 nm^−1^ versus the response functions 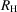 and 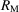 for *A* = 4.7 pJ m^−1^ and experimental field values (in mT) of 1270, 312, 103, 61, 42, 33. The plane represents a fit to equation (15). The intercept of the plane with the 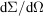 axis provides the residual SANS cross section 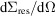 , while 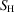 and 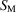 are obtained from the slopes of the plane (slopes of the thick black and red lines). In other words, at each experimental 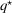 , for given materials parameters *A* and 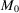 , and for the experimental field values 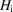 , the total experimental SANS signals at 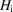 are fitted to a function that is of the mathematical form 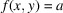 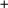 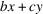 , where 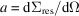 , 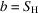 and 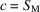 are the fit parameters at 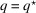 and 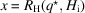 and 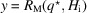 are the independent variables. The procedure is carried out for 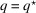 values between 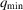 and 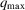 , and then repeated for many different physically plausible *A* values to determine the best-fit value, 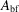 , via equation (25). Image taken from Michels (2021 ▸), reproduced by permission of Oxford University Press.

Equation (15) is linear in both 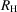 and 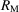 , with *a priori* unknown functions 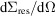 , 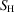 and 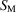 . For given values of the materials parameters *A* and 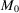 , the numerical values of both response functions are known at each value of *q* and 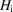 . By plotting at a particular 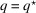 the values of 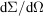 measured at several 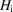 versus 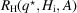 and 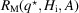 , one can obtain the values of 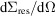 (intercept) and 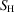 and 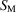 (slopes) at 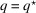 by a weighted non-negative linear least-squares plane fit (*i.e.* the parameters 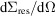 , 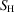 and 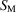 are assumed to be 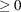 ). The function ‘lsqnonneg’ of MATLAB has been used for carrying out these fits. Starting from 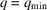 , the non-negative least-squares fitting routine is successively performed up to a maximum value of 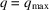 [see equation (24) below]. Fig. 2 ▸ illustrates the data analysis procedure. By treating the exchange-stiffness constant *A* in the expression for 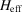 as an adjustable parameter, one can obtain information on this quantity. We emphasize that in order to obtain a best-fit value for *A* from experimental field-dependent SANS data, it is not necessary that the data are available in absolute units. This is because *A* only appears in the dimensionless response functions 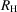 and 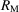 , while the dimension of the experimental 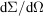 (in cm^−1^ or in arbitrary units) is absorbed in the other fitting parameters 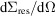 , 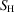 and 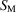 .

As mentioned earlier, the effective magnetic field 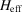 [equation (10)] is the sum of the internal magnetic field 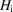 and the exchange field 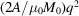 . When 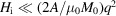 , the effective field and, hence, the magnetic SANS cross section become independent of the externally applied magnetic field 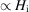 . This condition defines a characteristic maximum *q* value, 

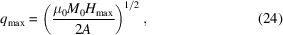

where 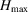 is the maximum applied magnetic field. For 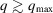 , the reliable separation of the spin-misalignment ( 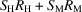 ) and residual scattering ( 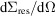 ) is difficult (since then one attempts to fit a straight line to a constant), and the micromagnetic analysis should therefore be restricted to 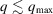 .

The global fitting procedure consists essentially of many straight-plane fits (one at each *q* value for 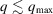 ). As the experimental best-fit parameter we take the value of *A* that minimizes the function 

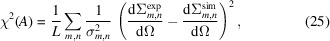

where the indices *m* and *n* count, respectively, the scattering vectors and applied-field values, *L* is the number of data points (number of *q* values times the number of internal fields), 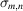 is the uncertainty in the experimental SANS cross section 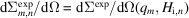 , and 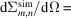 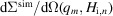 denotes the fit to equation (15) or (17).

The uncertainty 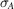 in *A* is estimated from the curvature of the 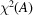 data, according to (Bevington & Robinson, 2003 ▸)

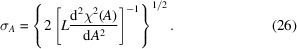

The numerical derivative in equation (26) has been computed via (Fornberg, 1988 ▸)

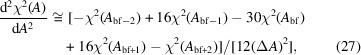

where 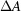 is the step size on the *A* axis (typically 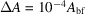 ), 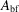 represents the global minimum of the function 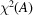 , 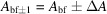 and 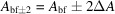 .

## 3. Description of the software

**Figure 3:**
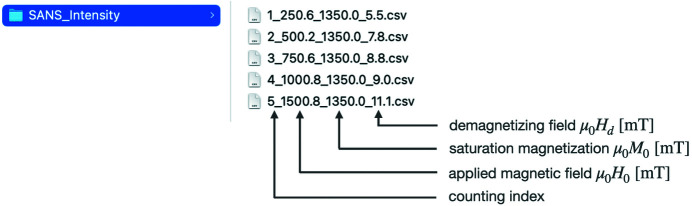
Explanation of the input data filename format. The specified numerical values for the applied magnetic fields 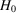 , saturation magnetization 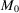 and demagnetizing fields 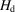 are automatically taken over by the *MuMag2022* software for the data analysis.

The least-squares fitting routine has been written in MATLAB code and implemented into a Windows- and macOS-compatible standalone executable file using the MATLAB app designer. The user has to provide the following data and take the following points into account:

(i) The total (nuclear and magnetic) unpolarized SANS cross section 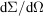 measured at several applied magnetic fields within the approach-to-saturation regime ( 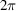 azimuthally averaged data). Data format: three columns with *q* in nm^−1^, 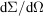 in cm^−1^ and the uncertainty in 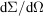 in cm^−1^. The input data files must be of the .csv, .dat or .txt type and must have the name structure that is explained in Fig. 3 ▸.

(ii) If the 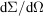 data are not available in absolute units, then the mean-square magnetic anisotropy field 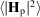 and magnetostatic field 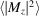 [equations (20)–(22)] cannot be determined. It is then only possible to estimate an average value for the exchange-stiffness constant *A*.

(iii) The values of the applied magnetic fields 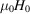 (in mT), where the SANS measurements have been carried out [see point (i) above]. Note that the quantities 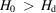 , 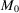 and 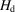 have the SI unit A m^−1^, which on multiplication with 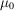 turns into Tesla (T).

(iv) The value of the saturation magnetization 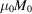 (in mT) of the sample [see point (i) above].

(v) The values of the demagnetizing fields 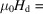 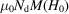 (in mT) [see point (i) above]. Note that in equation (11) the demagnetizing field was specified as 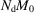 with 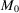 the saturation magnetization. The user may, however, take a different value of the demagnetizing field at each value of the externally applied magnetic field 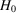 with corresponding magnetization value 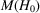 . The demagnetizing factor 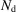 can be calculated using *e.g.* the well known formulas for the general ellipsoid by Osborn (1945 ▸) or for rectangular prisms by Aharoni (1998 ▸).

(vi) The data analysis should be restricted to internal magnetic fields 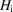 within the approach-to-saturation regime. This information can be taken from an experimental magnetization curve 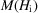 , which also allows for the determination of 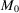 . We suggest defining ‘approach-to-saturation’ for 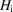 values for which the reduced magnetization is 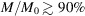 .

(vii) An estimate for 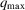 using equation (24). Typical *A* values are of the order of 10 pJ m^−1^ (1 pJ m^−1^ = 10^−12^ J m^−1^). The data analysis should be restricted to 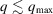 .

(viii) The following output files are generated (in .csv format). For the perpendicular scattering geometry ( 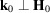 ): best-fit results (using 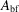 ) for the discrete functions 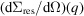 , 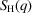 , 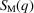 , 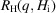 , 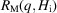 , 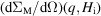 = 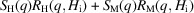 and 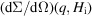 = 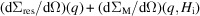 . For the parallel scattering geometry ( 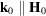 ): best-fit results (using 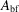 ) for the discrete functions 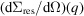 , 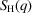 , 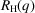 , 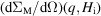 = 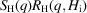 and 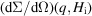 = 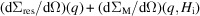 . Data format: three columns with *q* in nm^−1^, the respective quantity in cm^−1^ (if the input data are in absolute units) and the uncertainty in the respective quantity in cm^−1^. Note that 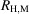 are dimensionless, while 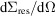 and 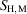 may be in cm^−1^. Moreover, for each scattering geometry, we specify the data set 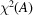 [equation (25)], the best-fit value for the exchange-stiffness constant 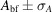 (in pJ m^−1^) [equation (26)], the root-mean-square anisotropy field 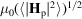 (in mT) and the root-mean-square magnetostatic field 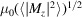 (in mT, only for 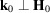 ). The provided data give the user the possibility to generate their own graphical representations.

## 4. Example cases

**Figure 4:**
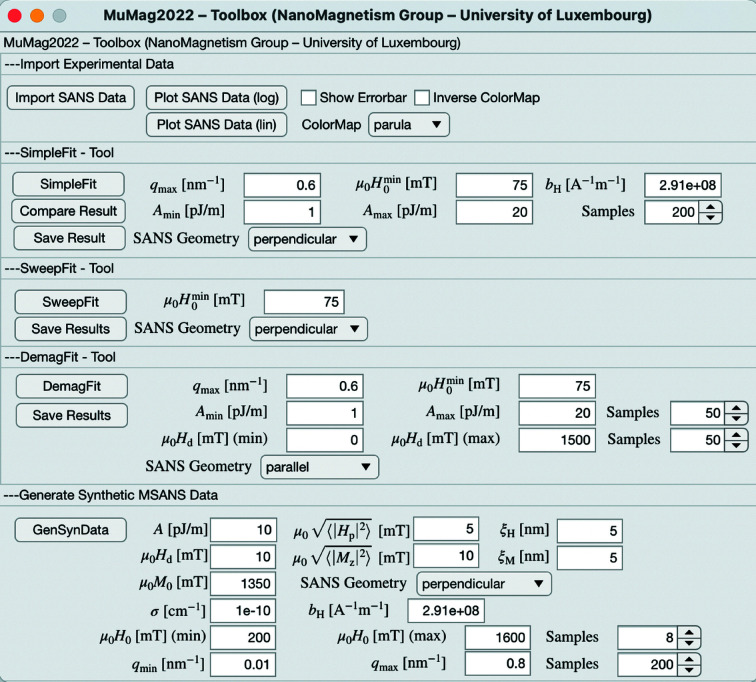
The user interface of the *MuMag2022* software.

The following example data on the two-phase iron-based alloy Nanoperm are taken from the work of Honecker *et al.* (2013 ▸), and the data on the Nd–Fe–B nanocomposite are those of Bick *et al.* (2013 ▸). Further examples in the literature where this type of SANS data analysis has been employed can be found in the work of Bersweiler *et al.* (2022 ▸) on another type of Nanoperm sample, and Weissmüller *et al.* (2001 ▸) and Michels *et al.* (2003 ▸) on nanocrystalline cobalt and nickel. Fig. 4 ▸ displays the user interface of the *MuMag2022* software, which is structured into five panels: (i) The top panel controls import and graphical representation of the experimental SANS data. (ii) For the selected scattering geometry ( 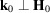 or 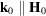 ), minimum applied field 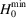 and maximum scattering vector 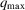 , the ‘SimpleFit’ tool determines the best-fit value 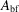 for the exchange-stiffness constant. (iii) The ‘SweepFit’ tool allows one to analyze the convergence of the fitting routine depending on the 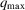 and 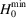 values. (iv) In case the demagnetizing field of the sample is unknown, the ‘DemagFit’ tool allows for the estimation of this quantity by additionally varying 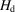 in the 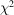 function [equation (25)]. The obtained best-fit values for *A* and 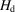 have then to be used in the ‘SimpleFit’ tool to generate the final fit results for 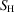 , 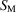 and 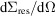 . (v) Finally, by specifying the scattering geometry, materials parameters, applied fields and *q* range, the *MuMag2022* software allows for the generation of synthetic data. We refer to the *MuMag2022–Toolbox: User Guide* for further details (https://files.uni.lu/mumag/MuMag2022_UserGuide.pdf).

**Figure 5:**
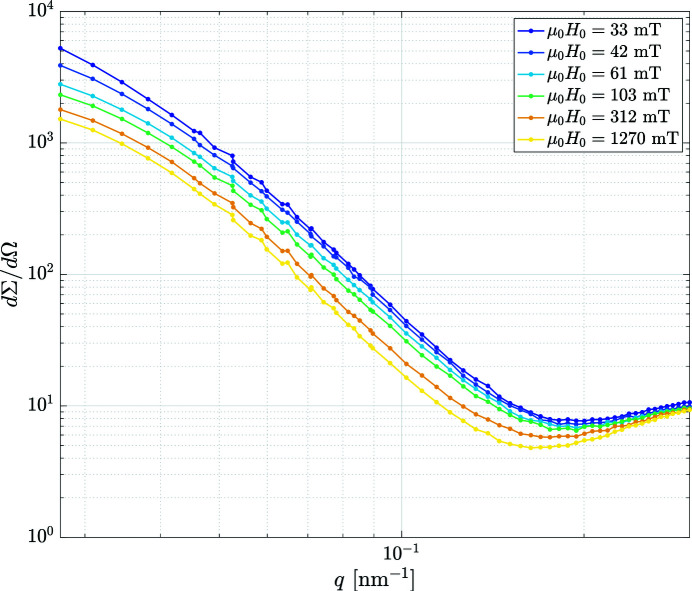
Total unpolarized experimental SANS cross section 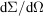 of the two-phase iron-based alloy Nanoperm at a series of applied magnetic fields (see legend) (log–log scale) ( 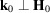 ). Lines are a guide for the eyes. Data taken from Honecker *et al.* (2013 ▸).

**Figure 6:**
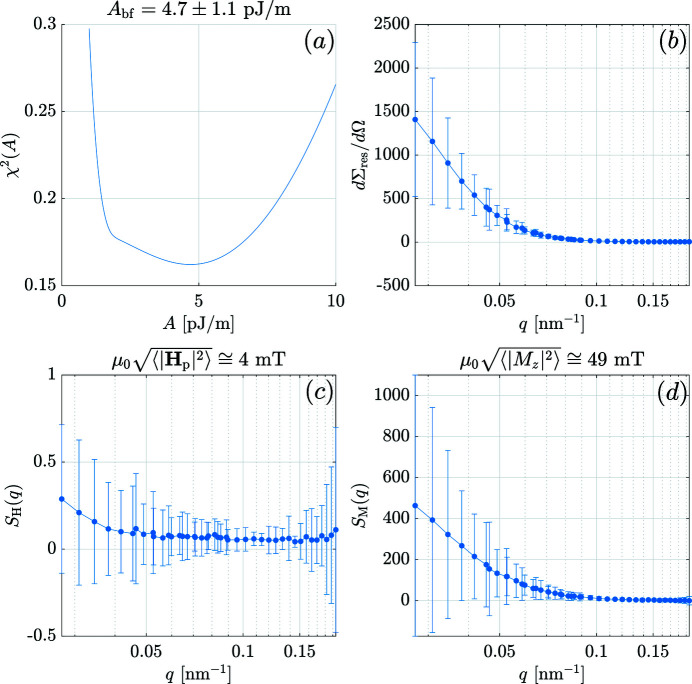
Summary of the fit results for Nanoperm. (*a*) 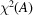 function [equation (25)]. (*b*) Residual SANS cross section 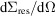 (linear–log scale). (*c*) Anisotropy-field scattering function 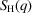 (linear–log scale). (*d*) Magnetostatic scattering function 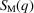 (linear–log scale). The best-fit value 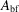 for the exchange-stiffness constant and the estimates for the mean anisotropy field 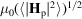 and the mean magnetostatic field 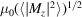 based on equation (20) are indicated. Settings from Fig. 4 ▸ in the user guide were used. Data taken from Honecker *et al.* (2013 ▸).

Figs. 5 ▸, 6 ▸, 7 ▸ have been exported from the *MuMag2022* software and show, respectively, the experimental field-dependent input data, the results of the data analysis, and the comparison between the experimental data and the fit based on the micromagnetic theory. Note that in Figs. 5 ▸ and 7 ▸ the values of the applied magnetic fields 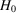 are displayed in the legends, while the internal magnetic fields 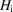 (using the values for 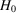 and 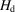 specified in the input data files) have been used for internal computations. The best-fit value for the exchange-stiffness constant of Nanoperm, 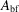 = 4.7 × 10^−12^ J m^−1^, is found from the minimum of the 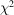 function in Fig. 6 ▸(*a*), while the *q* dependence of 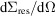 , 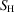 and 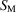 is featured in Figs. 6 ▸(*b*)–(*d*), respectively. The results for the average anisotropy ( 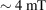 ) and magnetostatic ( 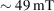 ) fields [Figs. 6 ▸(*c*) and 6 ▸(*d*), respectively] demonstrate that the strongest perturbations in the spin structure are related to the jumps in the saturation magnetization at internal particle–matrix interfaces, in agreement with the two-phase microstructure of the material.

**Figure 8:**
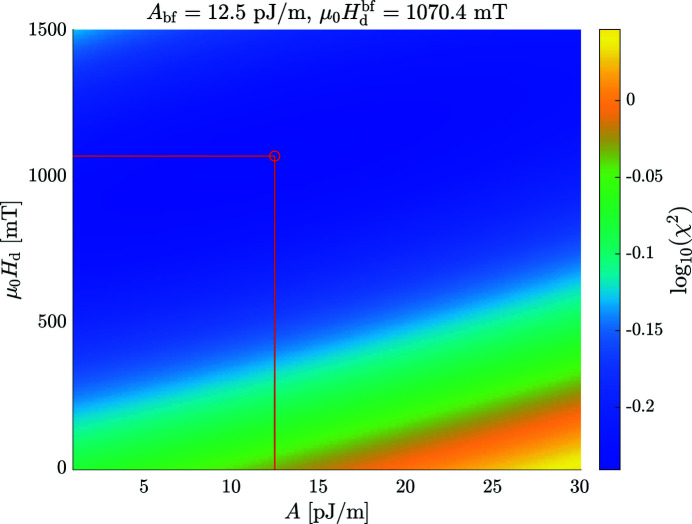
Pseudocolor plot of 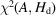 [equation (25)] for an Nd–Fe–B nanocomposite ( 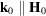 ). The best-fit values, 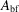 and 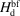 , are indicated. Data taken from Bick *et al.* (2013 ▸).

The *MuMag2022* software also allows for treating the demagnetizing field 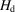 [in the expression for 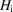 , compare equation (11)] as an adjustable parameter, *e.g.* in situations where the sample shape is not well defined. This is achieved by varying 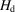 , in addition to *A*, within the limits 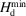 and 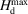 in the 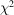 function [equation 25)]. Fig. 8 ▸ shows the output of the ‘DemagFit’ tool for the case of an Nd–Fe–B nanocomposite measured in the parallel scattering geometry ( 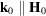 ).

The micromagnetic SANS theory on which *MuMag2022* is based assumes a statistically isotropic ferromagnetic material with random nanoscale variations in the magnitude and orientation of the magnetic anisotropy field as well as nanoscale spatial variations in the saturation magnetization. Recently, an extended SANS theory which takes into account a global uniaxial anisotropy (magnetic texture) has been developed (Zaporozhets *et al.*, 2022 ▸). The corresponding equations for the SANS cross sections will be implemented in a future version of *MuMag2022*.

## 5. Conclusion

The MATLAB-based software tool *MuMag2022* allows for the analysis of magnetic-field-dependent small-angle neutron scattering (SANS) data of bulk ferromagnets. Examples of such systems are elemental nanocrystalline ferromagnets, magnetic nanocomposites and magnetic steels. The software is based on the micromagnetic theory for the magnetic SANS cross section, and analyzes unpolarized total (nuclear and magnetic) SANS data within the approach-to-saturation regime of the macroscopic magnetization. The main features of *MuMag2022* are the estimation of the exchange-stiffness constant, and of the strength and spatial structure of the magnetic anisotropy field and the magnetostatic field due to longitudinal magnetization fluctuations. *MuMag2022* comes with a user-friendly interface and is available along with the example data as a standalone executable for Windows operating systems. It can be downloaded at https://mumag.uni.lu. Additionally, we provide a *MuMag2022–Toolbox: User Guide* that should enable the operation of the software.

## Figures and Tables

**Figure 7:**
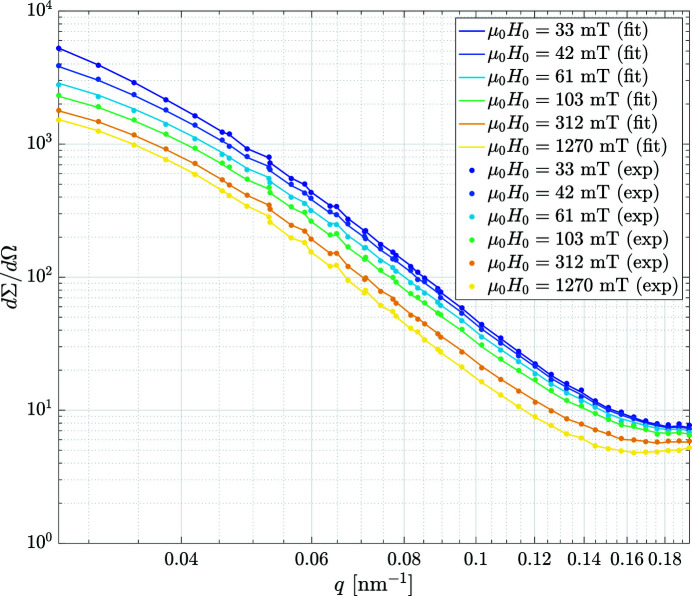
Comparison between experiment and theory. Data points: experimental data for the total unpolarized SANS cross section 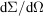 of the two-phase iron-based alloy Nanoperm at a series of applied magnetic fields within the approach-to-saturation regime (see legend) (log–log scale) ( 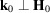 ). Solid lines: fit using the micromagnetic SANS theory [equation (15)] with the best-fit value of 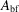 = 4.7 × 10^−12^ J m^−1^. The analysis has been restricted to fields 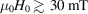 and to momentum transfers 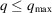 = 0.2 nm^−1^. Note that the fit does not represent a ‘continuous’ fit of 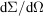 in the conventional sense, but rather the point-by-point reconstruction of the theoretical cross sections based on the experimental data. Data taken from Honecker *et al.* (2013 ▸).

## Footnotes

1 The acronym SANSPOL refers to a polarized SANS experiment without analysis of the polarization of the scattered neutrons.
